# Occupational Noise in the Central Sterile Supply Department: A Narrative Review of Exposure, Mechanisms, and Control

**DOI:** 10.3390/healthcare14152358

**Published:** 2026-08-03

**Authors:** Hai-Qin Zhang, Ping Cheng, Fu-Hai Ji, Ke Peng

**Affiliations:** 1 Central Sterile Supply Department, The First Affiliated Hospital of Soochow University, Suzhou 215006, China; 18662525017@163.com (H.-Q.Z.); chengping197307@suda.edu.cn (P.C.); 2 Department of Anesthesiology, The First Affiliated Hospital of Soochow University, Suzhou 215006, China; jifuhaisuda@163.com

**Keywords:** Central Sterile Supply Department, cognitive–affective mediation, occupational noise exposure, patient safety, quality management

## Abstract

The Central Sterile Supply Department (CSSD) functions as the critical control point for hospital infection prevention, yet its equipment-intensive environment generates occupational noise exposure that has been systematically overlooked in both the healthcare quality and occupational health literature. We conducted a focused narrative review across PubMed, Web of Science, and Scopus (up to March 2026) to examine the prevalence, psychophysiological mechanisms, and management of noise in CSSD settings. Four peer-reviewed studies investigating CSSD noise exposure were identified, conducted in Chinese and Brazilian hospitals. The evidence indicates that air guns and pressure steam sterilizers constitute the dominant noise sources, with self-reported exposure associated with psychological symptoms and sleep disturbance in approximately one-quarter of staff. Notably, health concerns may mediate a substantial proportion of the noise–psychology relationship, suggesting a cognitive–affective pathway that may be as theoretically significant as direct physiological damage, although this cross-sectional finding requires longitudinal confirmation. No study has empirically linked CSSD noise to sterilization failures, device damage, or patient-level outcomes. Drawing on this nascent evidence base and adjacent occupational noise theory, we advance three testable propositions: that CSSD noise may constitute a systemic quality risk factor, that subjective cognitive–affective appraisal plays a primary mediating role, and that effective management requires multi-level integration across engineering, administrative, and psychological domains. This review reframes CSSD noise from an ergonomic nuisance to an embedded environmental stressor and charts a structured research agenda for objective measurement, mechanism validation, and intervention evaluation.

## 1. Introduction

The Central Sterile Supply Department (CSSD), as the “master valve” of hospital infection control, manages the full-cycle processing of reusable medical devices, from cleaning and sterilization to sterile supply [1,2,3]. While research has extensively addressed output quality indicators such as sterilization efficacy and infection rates [4,5,6,7], the occupational noise generated by core CSSD equipment, including air guns, pressure steam sterilizers, and automated washers, has received minimal attention. These devices operate at high intensity within enclosed, acoustically untreated spaces, creating sustained exposure conditions that may compromise the cognitive vigilance and psychological well-being of personnel.

Unlike operating rooms or intensive care units, where environmental stressors have been widely studied, the CSSD has historically been recognized as a support function, rendering its acoustic environment largely invisible in the healthcare literature [8,9,10,11,12]. With an estimated CSSD workforce of approximately 200,000 in China alone, this occupational exposure affects a substantial population of healthcare workers, yet it remains systematically unmeasured in both clinical and regulatory frameworks [13]. Emerging evidence indicates that CSSD noise exposure is associated with psychological symptoms, sleep disturbance, and reduced work competency [14,15]. However, the mechanisms linking noise to these outcomes, and the management strategies capable of interrupting these pathways, remain poorly characterized. 

Therefore, this narrative review addresses these gaps by synthesizing evidence across three integrated domains: (i) the epidemiology and acoustic characteristics of CSSD noise exposure; (ii) psychophysiological mechanisms linking noise to health and performance outcomes; and (iii) emerging noise reduction and quality management strategies.

## 2. Methodology

We conducted a focused literature search across PubMed, Web of Science, and Scopus (up to March 2026) using combinations of the following terms: “central sterile supply department”, “CSSD”, “sterile processing department”, “noise”, “occupational noise exposure”, “acoustic environment”, “work environment”, and “quality management”. No language restrictions were applied.

Inclusion criteria were (i) peer-reviewed studies investigating noise exposure, health outcomes, or work performance in CSSD or analogous sterile processing settings; (ii) management model evaluations that contextualized the CSSD work environment; and (iii) established theoretical frameworks from occupational health and environmental psychology literature. Exclusion criteria were (i) single-hospital quality improvement reports lacking methodological detail; (ii) non-peer-reviewed sources (such as editorials, conference abstracts, and preprints); (iii) studies confined to non-healthcare industrial settings without relevance to sterile processing; and (iv) publications focused exclusively on equipment engineering without human or organizational outcomes.

We adopted a narrative review approach to synthesize available evidence and construct a preliminary conceptual framework. Four peer-reviewed studies directly investigating CSSD noise exposure were identified [14,15,16,17]. To ensure methodological transparency, we note the following: These three databases were selected because they comprehensively capture the peer-reviewed biomedical (PubMed) and multidisciplinary (Web of Science and Scopus) literature in which eligible studies were most likely to be indexed; preliminary scoping suggested that additional international databases were unlikely to yield further eligible studies given the extreme paucity of literature in this specific niche. Chinese databases were not searched because preliminary scoping indicated that CSSD noise publications in Chinese journals consist almost exclusively of single-hospital quality-improvement reports, which fall under the exclusion criterion (i). Reference lists of all included studies were hand-searched, and backward–forward citation tracking was performed to minimize omission risk. Given the narrative design, a formal risk-of-bias tool was not applicable; instead, each included study was critically appraised for design, exposure assessment, and key limitations (Table 1), and these appraisals inform the cautious interpretation offered throughout this review.

## 3. The CSSD Acoustic Environment

### 3.1. Noise Sources and Characteristics

The CSSD concentrates multiple high-intensity noise sources within a workflow that demands continuous staff proximity. In a cross-sectional survey of 308 CSSD staff across 30 hospitals in China, Wang et al. identified two dominant sources: air gun sounds, reported as troublesome by 76.3% of respondents, and pressure steam sterilizer noises (78.2%) [14]. These sources are intrinsic to the core workflow: air guns for drying and cleaning verification and steam sterilizers as the primary sterilization modality worldwide.

Objective dosimetry data specific to CSSDs remain scarce. Healthcare processing environments (central laundry, automated instrument reprocessing) report peak noise levels of 85–95 dB(A) during equipment operation cycles, frequently exceeding the 85 dB(A) threshold recommended by the World Health Organization for 8-hour occupational exposure. Sterilizer venting and air gun discharge produce impulsive noise profiles whose high sound energy, rather than any specific high-frequency feature, is the principal reason such stimuli can exceed the protection offered by conventional passive earplugs. 

Beyond A-weighted sound pressure levels, a complete characterization of the CSSD acoustic environment requires temporal variability, impulsiveness (for example, crest factor or kurtosis), spectral content, and room reverberation, which jointly determine annoyance, speech interference, and physiological response; to date, no CSSD study has reported descriptors beyond A-weighted levels or self-reported perception. Exposure assessments should also state the risk criterion applied: for personal dosimetry, a 3 dB exchange rate (Q = 3), consistent with the equal-energy principle and recommended by the National Institute for Occupational Safety and Health, offers greater worker protection than the 5 dB rate adopted in some regulatory frameworks [18].

The physical layout of CSSDs further compounds exposure. Standard unidirectional flow designs (dirty receipt, washing, packaging, and sterile storage) theoretically separate the noisiest equipment from quieter zones [3]. In practice, acoustic barriers between zones are often inadequate, particularly in older facilities or resource-constrained settings. Staff rotation across zones during single shifts results in intermittent but repeated exposure to high-intensity bursts, a pattern that personal noise dosimetry studies in other healthcare settings suggest may be more physiologically disruptive than continuous low-level noise.

### 3.2. Prevalence and Exposure Patterns

Epidemiological evidence indicates that CSSD noise exposure is both highly prevalent and chronically underrecognized. Complementary evidence from Brazil and China confirms the international generalizability of these findings. Da-Silva et al. employed an ergonomic analysis of work in a northern Brazilian hospital’s CSSD and found that workers were exposed to noise coming from autoclaves, central air conditioning, and medicinal gas [17]. This study also identified inappropriate lighting as a co-occurring environmental hazard, suggesting that CSSD noise exposure operates within a broader constellation of physical environmental stressors that may exert synergistic effects.

Wang et al. found that approximately 25.3% of CSSD staff reported significant sleep disturbance attributed to workplace noise, and 32.1% expressed serious concerns about noise-related health effects [14]. In China, noise is the leading occupational health hazard, affecting 72% of workers across industries; globally, 28% of European workers report noise exposure for at least one-quarter of working hours [14]. The CSSD sits within a regulatory blind spot: healthcare settings are frequently exempt from industrial noise regulations, and CSSD-specific exposure limits do not exist in any national jurisdiction. Consequently, noise in this department is systematically unmeasured, unmanaged, and absent from hospital accreditation standards.

Critical limitations of the current evidence base must be acknowledged. The majority of CSSD noise studies identified to date were conducted in Chinese tertiary hospitals, with emerging evidence from Brazil. Generalizability to other healthcare systems remains unknown. Furthermore, no study has deployed continuous personal noise dosimetry throughout full shifts; existing data rely on self-report or spot measurements, precluding precise dose–response estimation.

## 4. Psychophysiological Mechanisms

The pathways described below are inferred from cross-sectional associations and established theoretical frameworks in occupational health psychology. They represent hypothesized rather than confirmed causal mechanisms in the CSSD context.

### 4.1. The Stress–Health Pathway

The available evidence suggests that noise operates as a chronic environmental stressor affecting physiological, psychological, and cognitive domains concurrently. Wang et al. reported that perceived noise exposure was associated with psychological symptoms (odds ratio [OR] = 1.57, 95% confidence interval [CI] = 1.04, 2.48), alongside age (OR = 1.06, 95% CI = 1.01, 1.11), educational background (OR = 0.65, 95% CI = 0.49, 0.85), health concerns (OR = 1.91, 95% CI = 1.06, 3.52), and self-reported hearing loss (OR = 1.46, 95% CI = 1.02, 2.10) [14]. Because these data are cross-sectional, directionality cannot be assumed: psychological distress may heighten noise perception, or unmeasured confounders (e.g., overall workplace adversity) may drive the association.

### 4.2. Health Concerns as a Cognitive–Affective Mediator

The most theoretically intriguing finding is the pattern consistent with a mediation structure linking noise to psychological outcomes through health concerns. Wang et al. reported a mediation effect value of 0.11 (95% CI = 0.08, 0.16) and an effect ratio of 53.8% [14]. This suggests that a substantial proportion of the noise-related psychological burden may be transmitted through worry about one’s health rather than direct physiological damage. However, this interpretation remains speculative because the mediation analysis was conducted on concurrent measurements. Health anxiety may amplify noise annoyance, or both may reflect an underlying trait of neuroticism.

Nevertheless, this hypothesized pathway aligns with cognitive appraisal frameworks in occupational stress research. It raises two testable propositions: (i) interventions targeting noise reduction alone may be insufficient if health anxiety is not addressed, and (ii) the perception of noise as dangerous may be as consequential as its objective decibel level.

### 4.3. Work Competency and Process Reliability

Beyond psychological well-being, the noise–performance nexus represents a critical concern for patient safety. Wu et al. investigated the effect of noise interference on work competency and coping styles among specialist nurses in CSSDs, finding that noise exposure significantly influenced both the professional competency and the adaptive strategies [15]. This study extends the theoretical model from individual health outcomes to organizational performance, suggesting that CSSD noise may compromise the very processes the department exists to execute. 

No study has directly linked CSSD noise to sterilization failures, device damage, or nosocomial infections. However, if noise degrades attention and precision, it may first compromise process reliability (e.g., inconsistent protocol adherence) before culminating in measurable quality defects. The CSSD quality management literature has developed numerous quality-sensitive indicators, yet none incorporate noise as a variable affecting process reliability. This omission represents a critical theoretical gap.

## 5. Management Interventions

### 5.1. Noise-Specific Management Programs

Evidence for noise-specific interventions in CSSDs is limited to a single peer-reviewed study. Wei et al. evaluated a structured noise reduction management program in one hospital CSSD, reporting reduced environmental noise levels and decreased auditory fatigue among staff after implementation [16]. These findings are preliminary: the study employed a before-after design without concurrent control, with short follow-up and outcomes restricted to auditory fatigue. Whether such programs produce sustained improvements in psychological well-being or work competency remains unknown. Multi-component interventions addressing engineering, administrative, and psychological levels have not been evaluated in the CSSD setting. Importantly, evidence-based findings must be distinguished from theoretically recommended practices: to date, only this single-component program has been supported by direct CSSD evidence, whereas the multi-level and quality-framework strategies discussed below are extrapolated from general occupational noise management and should be read as theory-informed recommendations rather than established CSSD practice.

### 5.2. CSSD Quality Management Frameworks

The CSSD quality management literature has developed sophisticated indicator systems, yet none incorporate the acoustic environment as a structural variable. Wang et al. demonstrated that a subspecialty management model improved satisfaction scores (95.8 ± 1.2 vs. 90.2 ± 2.3, *p* < 0.001), reduced complaint rates (3.3% vs. 11.6%, *p* = 0.035), and lowered device damage rates (0.4% vs. 0.61%, *p* = 0.003) [19]. Jing et al. constructed quality-sensitive indicators under key-point control theory [5], and Hu et al. established nursing-sensitive quality indicators using the Structure–Process–Outcome model [20]. Subsequent work on visual management, whole-process quality control, and workload optimization has refined operational standardization [21,22,23]. 

Despite this evolution, the human factors dimension (cognitive, psychological, and physiological state of the workforce) has been treated as a constant rather than a variable. The acoustic environment remains absent from all identified quality frameworks, reflecting the dominant process-centric orientation of CSSD management research. The emerging noise literature challenges this assumption, but the two evidence bases have developed in parallel without theoretical intersection. The challenge Charleman explicitly addresses, including noise and electronic distraction and their impact on quality patient care within the central supply department, represents an emerging theoretical recognition that environmental factors must be integrated into CSSD quality frameworks rather than treated as exogenous to the quality management conversation [24]. 

## 6. Theoretical Synthesis

**Figure 1:**
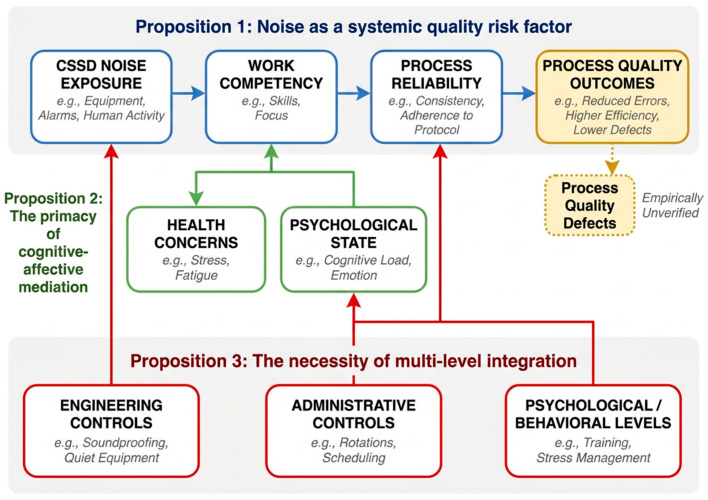
Preliminary conceptual framework proposing three testable propositions linking CSSD noise exposure to process quality outcomes. Notes: Proposition 1 posits that CSSD noise exposure may degrade work competency, which in turn compromises process reliability and, theoretically, culminates in process quality defects. Proposition 2 hypothesizes that the noise–psychology relationship operates through two interrelated cognitive–affective pathways—health concerns and psychological state—which together mediate the effect of noise on work competency. Proposition 3 posits that effective noise management requires multi-level integration across engineering, administrative, and psychological/behavioral controls. The pathway from noise to process quality defects remains empirically unverified.

Drawing together the evidence reviewed above, we propose a preliminary conceptual framework (Figure 1) advancing three testable propositions: proposition 1 establishes why noise matters for quality management; proposition 2 explains how it matters through psychological mechanisms; and proposition 3 specifies where interventions must operate. Collectively, they reframe CSSD noise from a peripheral ergonomic concern to a theoretically significant environmental stressor.

### 6.1. Proposition 1: Noise as a Systemic Quality Risk Factor

CSSD noise is not merely an occupational health issue but a latent pathway to process quality degradation that should be incorporated into quality management frameworks alongside traditional indicators such as sterilization monitoring and device tracking. The current process-centric orientation of CSSD management treats workforce capacity as a constant; we suggest that environmental stressors may function as structural variables affecting process reliability. This proposition does not claim that noise directly causes sterilization failures; rather, it posits that the acoustic environment constitutes an unmeasured source of variance in quality outcomes that existing frameworks ignore.

### 6.2. Proposition 2: The Primacy of Cognitive–Affective Mediation

The finding that health concerns may mediate a substantial proportion of the noise–psychology relationship suggests that subjective appraisal processes are as theoretically important as objective exposure levels. If confirmed by longitudinal designs, this would imply that interventions must target both the physical environment and the psychological interpretation of that environment. The perception of noise as dangerous may be as consequential as its decibel level, a hypothesis derived from general occupational stress theory but untested in CSSD contexts.

### 6.3. Proposition 3: The Necessity of Multi-Level Integration

Effective noise management in the CSSD cannot be achieved through equipment-level fixes alone. It requires integration across facility design (architectural acoustics), operational management (scheduling to minimize cumulative exposure), workforce development (coping resources and health literacy), and equipment engineering (modernization of noise-generating machinery). This proposition is currently supported by inference rather than direct evidence, as no multi-level intervention has been evaluated in the CSSD setting.

## 7. Future Research Agenda

The nascent state of this literature demands a structured research agenda organized around the theoretical tensions and gaps identified above. 

### 7.1. Measurement and Benchmarking

Proposition 1 requires objective validation. The field currently lacks standardized protocols for measuring noise exposure in the CSSD. Future studies should deploy continuous personal noise dosimetry throughout full shifts alongside subjective perception measures to establish dose–response relationships specific to the CSSD acoustic environment. Cross-national comparisons are urgently needed, as the existing evidence base is confined to Chinese tertiary hospitals; generalizability to other healthcare systems remains unknown. Regulatory benchmarking is equally critical: no jurisdiction currently mandates CSSD-specific exposure limits, and hospital accreditation standards do not include acoustic environment assessment. Measurement protocols should report the risk criterion applied, preferably a 3 dB exchange rate (Q = 3) [18], and should capture descriptors beyond time-averaged A-weighted levels, including temporal variability, impulsiveness, spectral content, and room reverberation characteristics. 

### 7.2. Mechanism Elucidation

Proposition 2 demands longitudinal and experimental designs. The temporal dynamics of the noise/health concerns/psychological symptoms pathway are unknown: does chronic exposure progressively amplify health anxiety, or does adaptation occur? Furthermore, the cognitive performance pathway (noise–attentional disruption–error proneness–quality deficits) has been theoretically proposed but not empirically tested in the CSSD context. Experimental or quasi-experimental designs using simulated CSSD tasks under varying noise conditions would be particularly valuable. Critically, such studies must distinguish between the effects of objective decibel levels and subjective noise annoyance, as our synthesis suggests that cognitive–affective appraisal may mediate a substantial proportion of the impact.

### 7.3. Integration with Quality Management Theory

Proposition 1 requires testing whether incorporating noise-related indicators into existing quality-sensitive frameworks improves predictive validity for outcomes such as sterilization failures, device damage, or staff turnover. The CSSD quality management literature has developed sophisticated indicator systems; the question is whether adding environmental stressors as structural variables enhances their explanatory power. This integration will also face practical constraints: quality management systems are already burdened with multiple competing indicators, and the incremental value of acoustic variables must be demonstrated against implementation costs.

### 7.4. Intervention Design and Evaluation

Proposition 3 requires rigorous evaluation of multi-component interventions that simultaneously address engineering, administrative, and psychological levels. The preliminary findings of Wei et al. require replication with rigorous controlled designs, longer follow-up periods, and outcome measures that extend beyond auditory fatigue to encompass psychological well-being, work competency, and process quality indicators. Multi-component interventions that simultaneously address engineering controls (equipment), administrative controls (scheduling, zoning), and psychological support (coping resources) should be evaluated against single-component approaches. Given the resource constraints typical of hospital CSSDs, cost-effectiveness analyses will be essential for translation into practice. 

### 7.5. Intelligent Monitoring and Dynamic Scheduling

The ongoing digitalization of CSSDs opens possibilities for real-time noise monitoring integrated with workflow management systems. Whether such systems can dynamically adjust staff scheduling to minimize cumulative exposure (for instance, by rotating personnel through high-noise and low-noise tasks) remains untested. However, the feasibility of this approach depends on CSSD staffing levels, which are frequently inadequate to permit flexible rotation without compromising throughput.

## 8. Conclusions

The CSSD faces a structural tension: its infection control mission demands an equipment-intensive environment that generates noise conditions undermining the workforce capacities required for error-intolerant sterile processing. This review reframes this acoustic environment as a systemic risk factor, proposing that workforce psychophysiological state is an integral determinant of process reliability. The evidence base remains nascent, with objective dosimetry, causal mechanisms, and patient-level outcomes largely unexamined, underscoring how thoroughly CSSD noise has been neglected. This review illuminates this gap and charts research pathways to close it. 

The next concrete steps are (i) multicenter studies deploying full-shift personal dosimetry with standardized acoustic descriptors to establish exposure benchmarks; (ii) longitudinal testing of the hypothesized health-concern mediation pathway; (iii) controlled evaluations of engineering, administrative, and psychological–behavioral interventions, alone and in combination; and (iv) incorporation of acoustic indicators into CSSD quality frameworks and hospital accreditation standards. The ultimate goal is to make the CSSD acoustic environment a measured, managed, and regulated determinant of both workforce health and sterile processing reliability. 

## Figures and Tables

**Table 1:** Characteristics of studies investigating noise exposure in the CSSD.

Study (Year)	Design	Sample	Noise Assessment	Key Findings	Critical Limitations
Wang et al. (2024) [14]	Cross-sectional survey	308 CSSD staff, 30 hospitals	Self-report (air gun 76.3%, sterilizer 78.2% troublesome)	Noise exposure associated with psychological symptoms (OR 1.57); health concerns mediated 53.8% of noise–psychology relationship	No objective dosimetry; single-province; self-report bias
Wu et al. (2025) [15]	Cross-sectional	Specialist nurses in CSSD	Noise interference scale (not objective dB)	Noise significantly associated with reduced work competency and altered coping styles	No objective noise measurement; no quality outcome data
Wei et al. (2026) [16]	Before-after (no control)	CSSD staff in one hospital	Environmental noise levels (pre/post)	Noise reduction program reduced environmental noise and auditory fatigue	No concurrent control; short follow-up; single-center; outcomes limited to auditory fatigue
Da-Silva et al. (2021) [17]	Ergonomic analysis	CSSD in one Brazilian hospital	Autoclaves, central air conditioning, medicinal gas	Noise exposure identified; inappropriate lighting as co-occurring environmental hazard	Single hospital; no health outcome data; observational ergonomic assessment

CSSD, Central Sterile Supply Department; OR, odds ratio.

## Data Availability

No new data were created or analyzed in this study. Data sharing is not applicable to this article.

## Abbreviations

The following abbreviations are used in this manuscript:

CSSD	Central Sterile Supply Department
OR	Odds ratio
CI	Confidence interval
